# Isoflurane Inhibits Dopaminergic Synaptic Vesicle Exocytosis Coupled to Ca_V_2.1 and Ca_V_2.2 in Rat Midbrain Neurons

**DOI:** 10.1523/ENEURO.0278-18.2018

**Published:** 2019-01-24

**Authors:** Christina L. Torturo, Zhen-Yu Zhou, Timothy A. Ryan, Hugh C. Hemmings

**Affiliations:** 1 Department of Anesthesiology, Weill Cornell Medicine, New York, NY 10065; 2 Department of Pharmacology, Weill Cornell Medicine, New York, NY 10065; 3 Department of Biochemistry, Weill Cornell Medicine, New York, NY 10065

**Keywords:** anesthesia, calcium, dopamine, exocytosis, neuropharmacology, synaptic transmission

## Abstract

Volatile anesthetics affect neuronal signaling by poorly understood mechanisms. Activation of central dopaminergic pathways has been implicated in emergence from general anesthesia. The volatile anesthetic isoflurane differentially inhibits glutamatergic and GABAergic synaptic vesicle (SV) exocytosis by reducing presynaptic Ca^2+^ influx without affecting the Ca^2+^-exocytosis relationship, but its effects on dopaminergic exocytosis are unclear. We tested the hypothesis that isoflurane inhibits exocytosis in dopaminergic neurons. We used electrical stimulation or depolarization by elevated extracellular KCl to evoke exocytosis measured by quantitative live-cell fluorescence imaging in cultured rat ventral tegmental area neurons. Using trains of electrically evoked action potentials (APs), isoflurane inhibited exocytosis in dopaminergic neurons to a greater extent (30 ± 4% inhibition; *p* < 0.0001) than in non-dopaminergic neurons (15 ± 5% inhibition; *p* = 0.014). Isoflurane also inhibited exocytosis evoked by elevated KCl in dopaminergic neurons (35 ± 6% inhibition; *p* = 0.0007), but not in non-dopaminergic neurons (2 ± 4% inhibition). Pharmacological isolation of presynaptic Ca^2+^ channel subtypes showed that isoflurane inhibited KCl-evoked exocytosis mediated exclusively by either Ca_V_2.1 (P/Q-type Ca^2+^ channels; 30 ± 5% inhibition; *p* = 0.0002) or by Ca_V_2.2 (N-type Ca^2+^ channels; 35 ± 11% inhibition; *p* = 0.015). Additionally, isoflurane inhibited single AP-evoked Ca^2+^ influx by 41 ± 3% and single AP-evoked exocytosis by 34 ± 6%. Comparable reductions in exocytosis and Ca^2+^ influx were produced by lowering extracellular [Ca^2+^]. Thus, isoflurane inhibits exocytosis from dopaminergic neurons by a mechanism distinct from that in non-dopaminergic neurons involving reduced Ca^2+^ entry through Ca_V_2.1 and/or Ca_V_2.2.

## Significance Statement

Despite their medical importance, the mechanisms of action of general anesthetics have not been fully elucidated. Isoflurane, a widely used volatile anesthetic, inhibits voltage-gated sodium channels and differentially inhibits synaptic vesicle exocytosis depending on neurotransmitter phenotype. Here, we show that in dopaminergic neurons of the ventral tegmental area isoflurane acts via a sodium channel-independent mechanism to inhibit synaptic vesicle exocytosis in proportion to reduced presynaptic Ca^2+^ flux mediated by Ca_V_2.1 and/or Ca_V_2.2, in contrast to its effects in non-dopaminergic neurons. These findings provide a synaptic mechanism for the observed role of reduced dopamine release in anesthetic-induced unconsciousness and implicate presynaptic Ca^2+^ channels of dopaminergic neurons as important targets of isoflurane.

## Introduction

General anesthetics are essential medicines that induce a reversible state of amnesia, unconsciousness, and immobility in the face of intensely painful stimuli. Despite their widespread use in modern medicine, their mechanisms of action are not well understood (Hemmings et al., 2005b). The amnestic, hypnotic, and immobilizing effects of anesthetics differ in dose dependence, neuroanatomical regions involved, and molecular targets consistent with multiple mechanisms working in parallel to produce the state of anesthetic-induced unresponsiveness (Brown et al., 2011). However, general anesthesia can produce serious adverse side effects, including cardiovascular, respiratory, and cognitive dysfunction. It is therefore critical to identify the anesthetic mechanisms relevant for both their on-target and off-target actions, with the ultimate goals of designing safer and more selective anesthetics and of using currently available anesthetics in a rational mechanism-based manner to maximize therapeutic ratio.

Volatile anesthetics such as isoflurane modulate synaptic and extrasynaptic neurotransmission through multiple postsynaptic targets, primarily by potentiating inhibitory GABA_A_ receptors and depressing excitatory glutamatergic transmission via ionotropic glutamate receptors (Rudolph and Antkowiak, 2004). However, the GABA_A_ receptor antagonist bicuculline does not antagonize isoflurane-induced immobility, indicating a role for other targets in this effect (Zhang et al., 2004). The presynaptic effects of volatile anesthetics are not as well characterized as their postsynaptic effects due to the small sizes of nerve terminals and technical limitations of conventional electrophysiological techniques in recording presynaptically. Nevertheless, considerable neurochemical and neurophysiological evidence indicates that volatile anesthetics directly inhibit neurotransmitter release (Hemmings et al., 2005a,b).

Synaptic vesicle (SV) exocytosis is tightly coupled to the amount of Ca^2+^ entering the presynaptic bouton (Wu et al., 2004), which is determined primarily by presynaptic voltage-gated ion channels (Na^+^, Ca^2+^, and K^+^ channels) and modulatory receptors. Isoflurane depresses action potential (AP) amplitude in axons and boutons, which results in downstream reductions in Ca^2+^ influx and neurotransmitter release (Wu et al., 2004; Hemmings et al., 2005a; Ouyang and Hemmings, 2005). Isoflurane also inhibits neurotransmitter release from isolated nerve terminals with greater potency from glutamatergic than from GABAergic terminals (Westphalen and Hemmings, 2003, 2006), consistent with neurotransmitter-specific presynaptic anesthetic mechanisms. The cellular and molecular bases of this synaptic selectivity are unclear.

Voltage-gated Ca^2+^ channels play an essential role in neurotransmission by mediating Ca^2+^ influx that is closely coupled to exocytosis. Presynaptic Ca^2+^ channels are possible targets for inhibition of neurotransmitter release by volatile anesthetics, and are also involved in producing myocardial depression and vasodilation leading to significant cardiovascular side effects (Lynch et al., 1981; Bosnjak et al., 1991). Synaptic transmission at most central nervous system synapses is mediated by multiple Ca^2+^ channel subtypes that are closely coupled to SV exocytosis, most prominently Ca_V_2.1 (P/Q-type Ca^2+^ channels) and Ca_V_2.2 (N-type Ca^2+^ channels; Wheeler et al., 1994; Wu et al., 1999). The degree of Ca_V_2.1 and Ca_V_2.2 involvement differ not only between different neuron classes (Murakami et al., 2002; Evans and Zamponi, 2006) but also between nerve terminals of the same afferent axon (Reid et al., 1997; Ariel et al., 2013). Reports of the effects of volatile anesthetics on specific Ca^2+^ channel subtypes are inconsistent (Hall et al., 1994; Study, 1994; White et al., 2005; Joksovic et al., 2009) such that the extent to which inhibition of Ca^2+^ channels contributes to inhibition of SV exocytosis remains unclear.

Recent work suggests that presynaptic Ca^2+^ channels are not the principal targets involved in the inhibition of glutamate and GABA release by volatile anesthetics (Westphalen et al., 2013). Despite their central roles in wakefulness and arousal (Monti and Monti, 2007), few studies have investigated anesthetic effects on aminergic neurons, which have distinct mechanisms of transmitter release (Liu et al., 2018). Electrical stimulation of dopaminergic neurons in the rat ventral tegmental area (VTA), one of the principal midbrain dopaminergic nuclei (Barrot, 2014), induces emergence from isoflurane anesthesia in rats (Solt et al., 2014). We sought to clarify the neurotransmitter selectivity and presynaptic targets of volatile anesthetics by investigating the effects of isoflurane, a representative halogenated ether anesthetic, on SV exocytosis from central dopaminergic neurons to test the hypothesis that isoflurane inhibits dopamine release by a mechanism distinct from that involved in non-dopaminergic neurons.

## Materials and Methods

### Reagents and solutions

Isoflurane was obtained from Abbott, ω-conotoxin GVIA and ω-agatoxin IVA from Alomone Labs, and 6-cyano-7-nitroquinoxaline-2,3-dione (CNQX) and (2*R*)-amino-5-phosphonovaleric acid (AP5) from Tocris. All other reagents were from Sigma-Aldrich. Rat vMAT2-pHluorin was kindly provided by Robert Edwards (University of California, San Francisco, CA), and mouse VAMP-mCherry was from [Timothy Ryan]. Tyrode’s solution (119 mM NaCl, 2.5 mM KCl, 2 mM CaCl_2_, 2 mM, MgCl_2_, 25 mM HEPES, and 30 mM glucose; pH 7.4) was used as the standard buffer in all experiments. The glutamate receptor antagonists CNQX (10 μM) and AP5 (50 μM) were added to Tyrode’s solution to block postsynaptic excitatory synaptic transmission. For single AP studies, Tyrode’s solution contained 4 mM CaCl_2_. Saturated stock solutions of isoflurane were prepared and diluted to 0.7 mM [two times the minimum alveolar concentration (2 MAC)] in Tyrode’s solution and into gas-tight glass syringes for focal perfusion onto imaged neurons through a 150-μm diameter polytetrafluoroethylene tube in the imaging chamber. Accounting for 10–20% loss, the predicted final concentration of isoflurane was 0.64 mM, which corresponds to 2 MAC in rats, a clinically relevant concentration equivalent to two time the ED_50_, corrected to the experimental temperature of 30°C (Franks and Lieb, 1993). Isoflurane was applied for 5 min before imaging to allow uptake and equilibration. At the conclusion of each experiment, a sample was taken from the chamber for analysis of delivered isoflurane concentration using a Shimadzu GC-2010 Plus gas chromatography with external standard calibration (Ratnakumari and Hemmings, 1998).

### Cell culture

Experiments were conducted according to protocols approved by the [Weill Cornell Medicine] Institutional Animal Care and Use Committee and conformed to National Institutes of Health Guidelines for the Care and Use of Animals. Glial monolayers were prepared from cerebral cortex as feeder layers for primary VTA neuron cultures from Sprague Dawley postnatal day 1 male and female rats (Charles River Laboratories) as described previously (Mena et al., 1997). After 7 d *in vitro* (DIV), neurons were transfected with vMAT2-pHluorin or VAMP-mCherry using a DNA-calcium phosphate coprecipitation protocol (Goetze et al., 2004; Jiang and Chen, 2006) modified to ensure low density transfection so that images could be obtained from a single neuron. Data were acquired from only one neuron per coverslip to avoid the contaminating and potentially irreversible effects of each drug treatment. Each experimental group contained coverslips from two to four different batches of primary neuron cultures to minimize artifacts due to differing culture conditions.

### Imaging SV exocytosis

Live-cell epifluorescence imaging employed a Zeiss Axio Observer microscope with images acquired using an Andor iXon^+^ CCD camera (model DU-897E-BV) and APs were evoked with 1-ms current pulses delivered via platinum-iridium electrodes. Depolarization with elevated K^+^ Tyrode’s solution (50 mM KCl substituted for 50 mM NaCl and buffered to pH 7.4) was used to evoke SV exocytosis independent of Na_v_ involvement (57). Elevated K^+^ Tyrode’s solution was applied onto imaged neurons using a pressurized injector (PDES System, ALA) for 4 s at 29 μl/s as the chamber was continuously perfused with Tyrode’s solution with or without added drugs. Fluorescence data were acquired as described, and total pool (TP) of SVs was identified by perfusion with Tyrode’s solution containing 50 mM NH_4_Cl (substituted for 50 mM NaCl and buffered to pH 7.4).

### Imaging calcium influx

VAMP-mCherry, a red fluorescent protein fused to VAMP (vesicle associated membrane protein), was used to identify synaptic boutons for Ca^2+^ imaging experiments. Transfected neurons were loaded with 7 μM Fluo-5F AM, incubated for 10 min at 30°C, and washed by superfusion with Tyrode’s solution for 15 min. Neurons were stimulated with a single AP 5 times at 2-min intervals during superfusion with Tyrode’s solution containing 2 mM Ca^2+^ with or without 2 MAC isoflurane.

### Immunocytochemistry

*Post hoc* immunolabelling with mouse anti-tyrosine hydroxylase (TH) monoclonal antibody (MAB318, Millipore) was used to identify dopaminergic neurons following live cell imaging. Fixed neurons were immunolabelled with either a 1:1000 dilution of Alexa Fluor 594 goat anti-mouse (for SV exocytosis experiments using vMAT2-pHluorin) or Alexa Fluor 488 goat anti-mouse (for Ca^2+^ imaging experiments). Imaged neurons were identified by coordinates on the coverslips and photographed.

### Image and statistical analysis

Fluorescence data were analyzed in ImageJ (http://rsb.info.nih.gov/ij) with a custom plug-in (http://rsb.info.nih.gov/ij/plugins/time-series.html). Transfected boutons were selected as regions of interest (ROIs) based on their response to 50 mM NH_4_Cl for SV exocytosis experiments or labeling with VAMP-mCherry for Ca^2+^ measurements. Each bouton was subjected to a signal-to-noise ratio (SNR) calculation based on its response to the first control electrical stimulation, and ΔF was calculated as the difference of the average intensities between F_peak_ and F_baseline_. Fluorescence intensity changes for Ca^2+^ measurements were normalized to baseline as ΔF/F: (F_peak_ – F_baseline_)/F_baseline_. Boutons with SNR > 5 were used in the analysis. Data are expressed as mean ± SD. To allow expression of inhibition or potentiation, drug effects are shown as a percentage of either TP or control response. Statistical significance was determined by paired or unpaired two-tailed or one-tailed Student’s *t* tests and by paired or unpaired one-way ANOVA with Tukey’s *post hoc* test, with *p* < 0.05 considered significant. Normality was assayed using the Shapiro–Wilk normality test. All statistical data are displayed in Table 1. Statistical analysis and graph preparation used GraphPad Prism v7.05 (GraphPad Software, Inc.).

## Results

**Figure 1.**
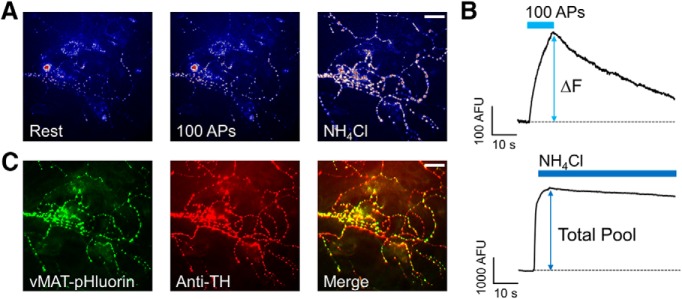
Measurement of SV exocytosis in dopaminergic neurons. ***A***, Representative fluorescence images of a cultured VTA neuron transfected with vMAT2-pHluorin at rest (left), after stimulation with a train of 100 APs delivered at 10 Hz (middle), and after perfusion with 50 mM NH_4_Cl in Tyrode’s solution (with an equivalent reduction in NaCl) to alkalinize the SV interior (right). Scale bar, 10 μm. ***B***, Representative traces of fluorescence responses to 100 APs at 10 Hz after 5 s of baseline fluorescence values (top), and to perfusion with 50 mM NH_4_Cl (bottom). Vertical arrow represents the change in fluorescence (ΔF). Blue bars indicate electrical stimulation (top) or NH_4_Cl perfusion that defines the TP (bottom). ***C***, Fluorescence images of a neuron transfected with vMAT2-pHluorin (green, left) and stained *post hoc* with anti-TH (red, middle). Composite image shows overlap of vMAT2-pHluorin and TH indicating this neuron is dopaminergic (right). Scale bar, 10 μm.

We used high resolution microscopy to quantify exocytosis at dopaminergic nerve terminals by the fluorescence change of pH-sensitive pHluorin fused to the luminal domain of the vesicular mononamine transporter vMAT2 (Anantharam et al., 2010; Pan and Ryan, 2012). Cultured rat midbrain neurons transfected with vMAT2-pHluorin were stimulated with trains of 100 APs at 10 Hz to elicit SV exocytosis (Fig. 1*A*). Increases in fluorescence (Δ*F*) following stimulation indicate alkalization of intravesicular pHluorin due to SV exocytosis. The difference between baseline and stimulus-evoked peak fluorescence reflects the amount of SV exocytosis; quenching of fluorescence in the post-stimulus period indicates SV endocytosis and re-acidification (Sankaranarayanan et al., 2000; Atluri and Ryan, 2006; Fig. 1*B*). The biosensor vMAT2-pHluorin reliably measured SV exocytosis over time with minimal decay in signal over the course of three control stimulations (stimulation 1 = 6.7 ± 0.9% of TP; stimulation 2 = 6.9 ± 0.9% of TP; stimulation 3 = 6.8 ± 0.9% of TP; *n* = 8; *p* = 0.93). Fluorescence data from each cell were normalized to the total SV pool defined by perfusion with 50 mM NH_4_Cl, which alkalizes the acidic SV interior and unquenches pHlourin fluorescence of the entire SV pool (Fig. 1*A*,*B*). Both dopaminergic and non-dopaminergic neurons can be transfected by vMAT2-pHluorin; dopaminergic neurons were positively identified by *post hoc* immunolabeling with mouse anti-TH (Fig. 1*C*).

### Isoflurane inhibits SV exocytosis in dopaminergic neurons

**Figure 2.**
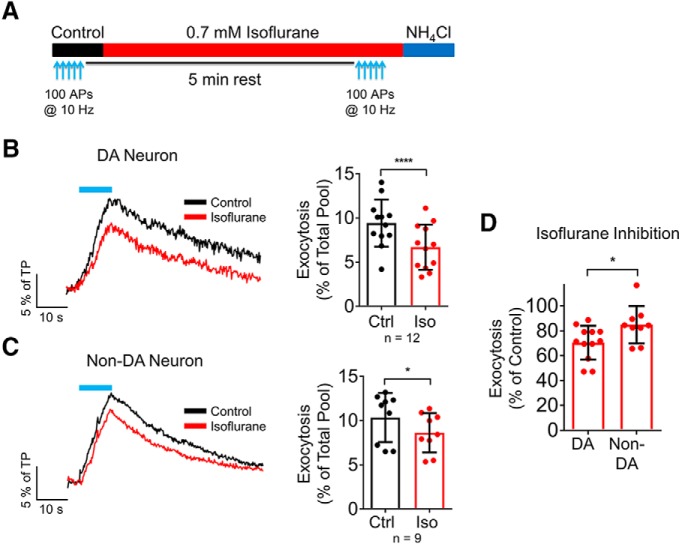
Isoflurane differentially inhibits SV exocytosis in dopaminergic and non-dopaminergic neurons. ***A***, Schematic of protocol used to assess the effect of isoflurane on SV exocytosis. 100 APs were delivered at 10 Hz (blue arrows) under control conditions, followed by perfusion with 0.7 mM (2 MAC) isoflurane for 5 min (red bar), and a second stimulation of 100 APs at 10 Hz in the presence of isoflurane. Lastly, NH_4_Cl was perfused to determine the TP (blue bar). ***B***, ***C***, Mean values of vMAT2-pHluorin response amplitudes in control and isoflurane-treated neurons stimulated with 100 APs at 10 Hz; ****p < 0.0001; *p < 0.05 by two-tailed paired *t* test. Representative raw traces from a dopaminergic (DA) and a non-dopaminergic (non-DA) neuron are shown. ***D***, The effect 0.7 mM isoflurane on 100 AP-evoked exocytosis was greater in dopaminergic than in non-dopaminergic neurons; *p < 0.05 by one-tailed *t* test.

Isoflurane at an immobilizing concentration (0.7 mM) inhibited SV exocytosis evoked by trains of 100 APs at 10 Hz in both dopaminergic (TH+) neurons and non-dopaminergic (TH–) neurons (Fig. 2). In dopaminergic neurons, control exocytosis was 9.4 ± 0.8% of TP, which was reduced to 6.7 ± 0.7% of TP by 0.7 mM (∼2× ED_50_) isoflurane (30 ± 4% inhibition; *p* < 0.0001; *n* = 12)^a^. In non-dopaminergic neurons, control exocytosis was 10.4 ± 0.9% of TP, which was reduced to 8.6 ± 0.7% of TP by isoflurane (15 ± 5% inhibition; *p* = 0.014; *n* = 9)^b^. The degree of inhibition of exocytosis was greater in dopaminergic neurons (*p* = 0.017; Fig. 2*D*)^c^. The time constant of pHluorin recovery was not significantly affected by isoflurane (DA Ctrl = 53 ± 15 s vs Iso = 45 ± 17 s; *n* = 12; non-DA Ctrl = 58 ± 11 s vs Iso = 46 ± 13 s; *n* = 9).

### Isoflurane effect is Na^+^ channel independent in dopaminergic neurons

**Figure 3.**
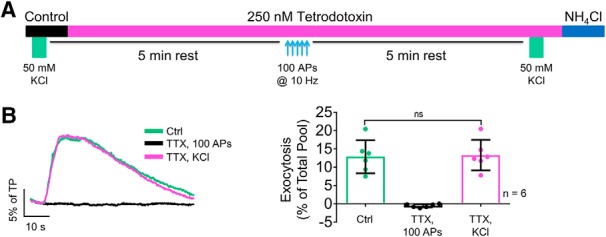
Elevated KCl depolarization-evoked SV exocytosis is Na_V_ independent. ***A***, Schematic of the protocol used to determine the effect of TTX on exocytosis evoked by elevated KCl or electrical stimulation. ***B***, Representative traces of vMAT2-pHluorin response to elevated KCl depolarization or electrical stimulation in the absence or presence of 250 nM TTX (left). Mean values of vMAT2-pHluorin response amplitudes (right). ns, not significant by paired one-way ANOVA with Tukey’s *post hoc* test.

**Figure 4.**
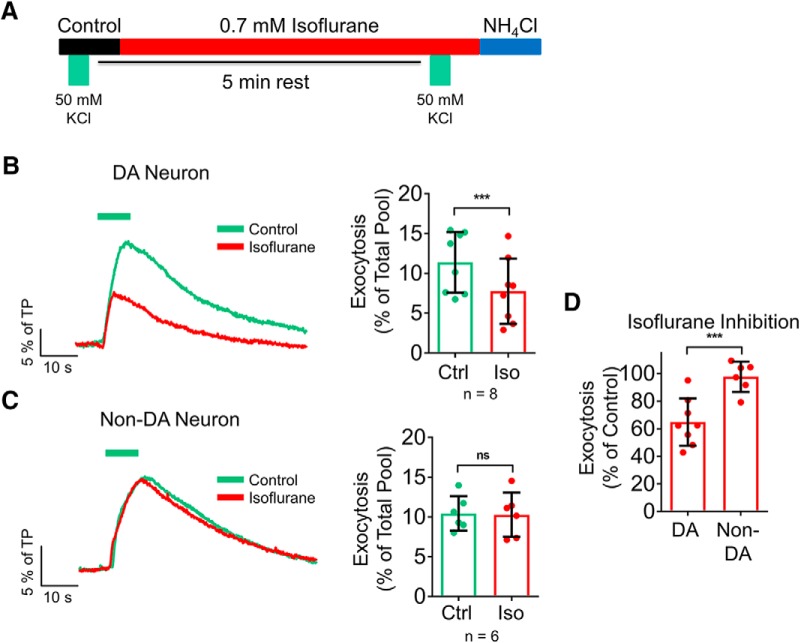
Isoflurane differentially inhibits SV exocytosis in dopaminergic neurons via a Na_v_-independent mechanism. ***A***, Schematic of protocol using a pulse of 50 mM KCl (with an equivalent reduction in NaCl, green bars) to induce exocytosis in the absence or presence of isoflurane. ***B***, ***C***, Mean values of vMAT2-pHluorin response amplitudes in control and 0.7 mM isoflurane-treated neurons stimulated with elevated KCl. Traces show representative raw traces from a dopaminergic (DA) and non-dopaminergic (non-DA) neuron; ***p < 0.001; ns, not significant by two-tailed paired *t* test. ***D***, Comparison of the effect of isoflurane on KCl-evoked exocytosis; ***p < 0.001 by one-tailed *t* test.

Ca^2+^-dependent SV exocytosis evoked by elevated extracellular KCl occurs by sustained depolarization that is independent of Na_V_ activation: it is insensitive to the specific Na_v_ blocker tetrodotoxin (TTX), in contrast to phasic AP-evoked SV exocytosis, which is completely blocked by TTX (Westphalen and Hemmings, 2003). Superfusion of TTX abolished SV exocytosis evoked electrically to –0.5 ± 0.2% of TP, yet had no effect on the response to elevated KCl (control with KCl = 12.9 ± 1.8% of TP, KCl with TTX = 13.3 ± 1.7% of TP; *p* = 0.92; *n* = 6; Fig. 3)^d^. We compared the effects of isoflurane on SV exocytosis evoked by elevated KCl, which directly activates Ca^2+^ channels, to SV exocytosis evoked electrically to determine whether isoflurane acts upstream or downstream of Ca^2+^ entry (Fig. 4*A*). We used a KCl concentration that evoked similar peak SV exocytosis compared to that obtained with the 100 AP stimulus train. Isoflurane inhibited elevated KCl-evoked exocytosis in dopaminergic neurons from 11.4 ± 1.3% of TP in control to 7.8 ± 1.5% of TP with isoflurane (35 ± 6% inhibition; *p* = 0.0007; *n* = 8; Fig. 4*B*,*D*)^e^. In contrast, isoflurane did not inhibit KCl-evoked SV exocytosis in non-dopaminergic neurons: 10.4 ± 0.9% of TP in control and 10.3 ± 1.1% of TP with isoflurane (2 ± 4% inhibition; *p* = 0.72; *n* = 6; Fig. 4*C*)^f^. Thus, isoflurane inhibited SV exocytosis in dopaminergic neurons by an Na_V_-independent pathway, in contrast to its Na_v_-dependent inhibition of SV exocytosis in non-dopaminergic neurons (Wu et al., 2004; Westphalen et al., 2013; Baumgart et al., 2015).

### Ca^2+^ channel subtypes mediating exocytosis in dopaminergic neurons

**Figure 5.**
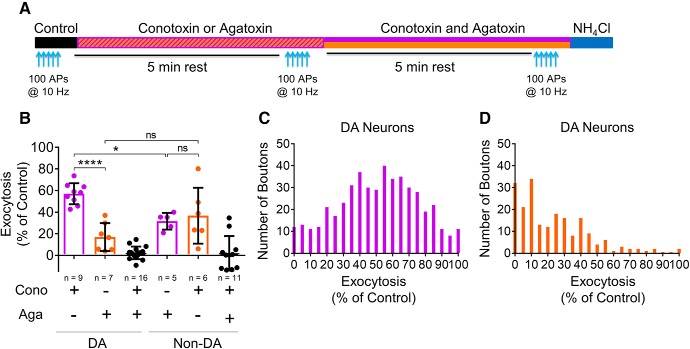
Ca_V_2.1 and Ca_V_2.2 contribute to SV exocytosis in VTA neurons. ***A***, Schematic of protocol using trains of 100 APs in the absence (black) or presence of ω-conotoxin GVIA (cono, 1 μM, purple bar) alone, ω-agatoxin IVA (aga, 400 nM, orange bar) alone, or both toxins together. ***B***, Comparison of the effect of conotoxin and agatoxin on dopaminergic and non-dopaminergic neurons. The combination of conotoxin and agatoxin abolished exocytosis in both dopaminergic (DA) and non-dopaminergic (non-DA) neurons; ****p < 0.0001; *p < 0.05; ns, not significant by unpaired one-way ANOVA, Tukey’s *post hoc* test. ***C***, Histogram displaying the bouton-by-bouton effect of conotoxin alone on exocytosis in dopaminergic neurons for all boutons (*n* = 9 neurons). Data have been constrained from 0% to 100% of control exocytosis. ***D***, Histogram displaying the bouton-by-bouton effect of agatoxin alone on exocytosis in dopaminergic neurons for all of boutons (*n* = 7 neurons). Data have been constrained from 0% to 100% of control exocytosis.

The role of specific Ca^2+^ channel subtypes in isoflurane inhibition of SV exocytosis was studied using the subtype-specific neurotoxin ω-conotoxin GVIA to block Ca_V_2.2 or ω-agatoxin IVA to block Ca_V_2.1 (Fig. 5*A*). Conotoxin alone inhibited SV exocytosis by 43 ± 3% (*n* = 9) in dopaminergic neurons and by 68 ± 3% (*n* = 5) in non-dopaminergic neurons, consistent with a greater contribution of Ca_V_2.2 in non-dopaminergic neurons than in dopaminergic neurons (*p* = 0.015; Fig. 5*B*)^g^. The Ca_V_2.1 blocker agatoxin alone inhibited SV exocytosis by 83 ± 5% (*n* = 7) in dopaminergic neurons and by 63 ± 11% (*n* = 6) in non-dopaminergic neurons, confirming a greater contribution of Ca_V_2.1 to SV exocytosis in dopaminergic neurons (conotoxin inhibition = 43 ± 3%; agatoxin inhibitio*n* = 83 ± 5%; *p* < 0.0001)^h^. In non-dopaminergic neurons, there was no significant difference in inhibition by conotoxin or agatoxin (*p* = 0.99)^i^, indicating similar contributions by both Ca_V_2.1 and Ca_V_2.2. There was no effect of the L-type Ca^2+^ channel inhibitor nimodipine (10 µM, Nimo) on SV exocytosis from dopaminergic or non-dopaminergic neurons (DA Ctrl = 7.8 ± 0.4% vs DA + Nimo = 7.2 ± 0.5% of TP, *n* = 7, *p* = 0.139; non-DA Ctrl = 10.1 ± 1.8% vs non-DA + Nimo = 9.8 ± 1.9% of TP, *n* = 5, *p* = 0.122). SV exocytosis in both dopaminergic and non-dopaminergic neurons was mediated exclusively by Ca_V_2.1 and Ca_V_2.2 since conotoxin and agatoxin together completely blocked SV exocytosis (Fig. 5*B*). A bouton by bouton analysis from all recorded neurons examined the effect of conotoxin (Fig. 5*C*) and agatoxin (Fig. 5*D*) in dopaminergic neurons and showed that the effects of the toxins correlated to the averaged effects (Fig. 5*B*).

### Isoflurane inhibits exocytosis mediated by Ca_V_2.1 and Ca_V_2.2

**Figure 6.**
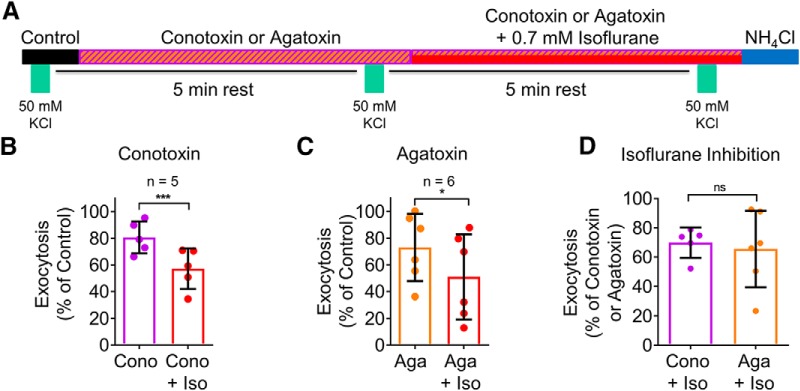
Isoflurane inhibits elevated KCl-evoked SV exocytosis mediated by Ca_V_2.1 or Ca_V_2.2 in dopaminergic neurons. ***A***, Schematic of protocol using depolarizing pulses of 50 mM KCl with 1 μM conotoxin and 0.7 mM isoflurane or with 400 nM agatoxin and 0.7 mM isoflurane in dopaminergic neurons. ***B***, Mean values of vMAT2-pHluorin response amplitudes in conotoxin and conotoxin + isoflurane treated dopaminergic neurons stimulated with elevated KCl; ***p < 0.001 by two-tailed paired *t* test. ***C***, Mean values of vMAT2-pHluorin response amplitudes in agatoxin and agatoxin + isoflurane treated dopaminergic neurons stimulated with elevated KCl; *p < 0.05 by two-tailed paired *t* test. ***D***, Comparison of the effect of isoflurane on Ca_V_2.1 and Ca_V_2.2 mediated elevated KCl-evoked exocytosis. ns, not significant by one-tailed *t* test.

We investigated the isoflurane sensitivity of SV exocytosis mediated by either Ca_V_2.1 or Ca_V_2.2 using pharmacological isolation (Fig. 6*A*). To examine the effect of isoflurane on Ca^2+^ channels without contributions by inhibition of upstream Na^+^ channels, we evoked exocytosis with elevated KCl depolarization. Conotoxin reduced KCl-evoked exocytosis to 81 ± 5% of control, and conotoxin plus isoflurane reduced exocytosis to 57 ± 7% of control (30 ± 5% inhibition; *p* = 0.0002, *n* = 5; Fig. 6*B*)^j^. A similar degree of isoflurane inhibition was obtained using 100 AP electrical stimulation with conotoxin and isoflurane (Cono = 8.3 ± 1.0% vs Cono + Iso = 5.4 ± 0.7% of TP, 35 ± 5% inhibition, *n* = 8, *p* < 0.001). Agatoxin reduced KCl-evoked exocytosis to 74 ± 10% of control, and agatoxin plus isoflurane reduced exocytosis to 51 ± 13% of control (35 ± 11% inhibition; *p* = 0.015; *n* = 6; Fig. 6*C*)^k^. There was no significant difference in the degree of isoflurane inhibition of Ca_V_2.1 versus Ca_V_2.2-mediated KCl-evoked exocytosis in dopaminergic neurons (*p* = 0.37; Fig. 6*D*)^l^.

### Isoflurane inhibits exocytosis by reducing Ca^2+^ entry

**Figure 7.**
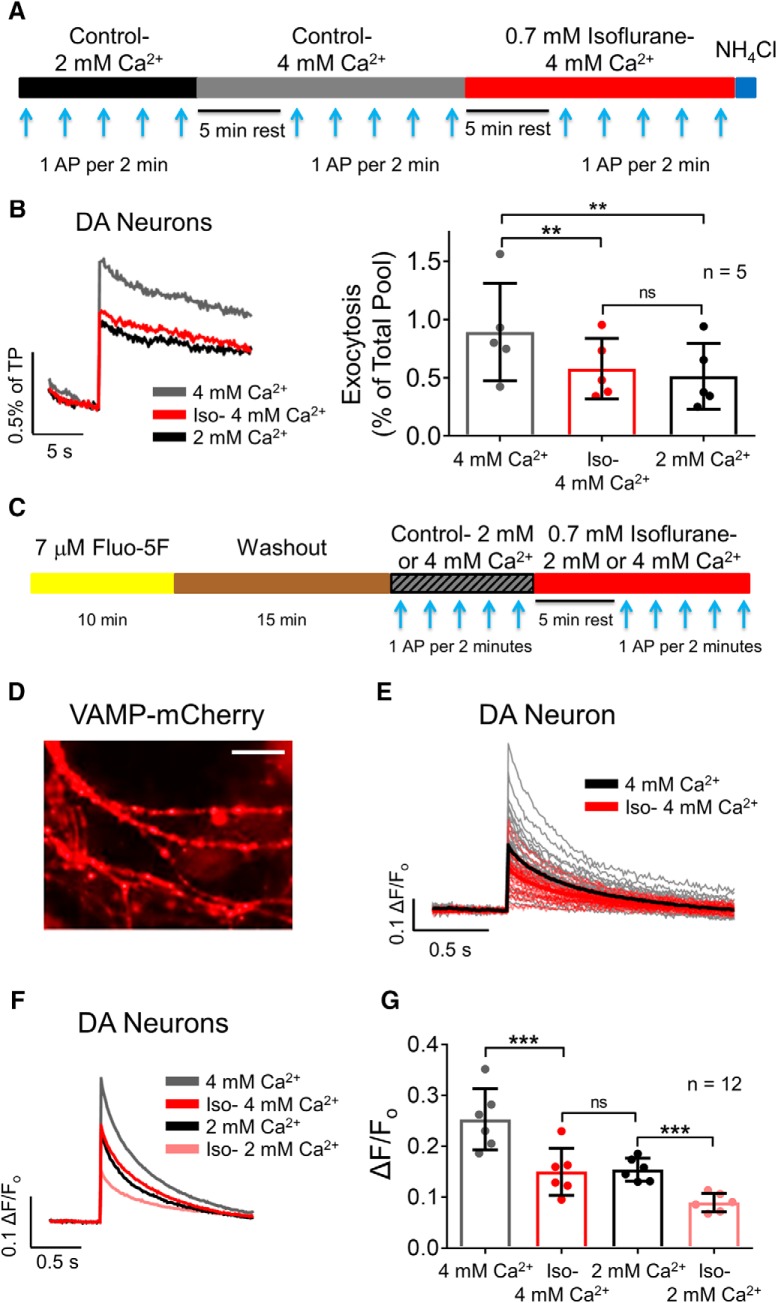
Isoflurane inhibits SV exocytosis in dopaminergic neurons by reducing Ca^2+^ influx. ***A***, Schematic of single AP (1 AP) evoked exocytosis protocol with 2 mM [Ca^2+^]_e_ (black bar), 4 mM [Ca^2+^]_e_ (gray bar), and 2 MAC isoflurane in 4 mM [Ca^2+^]_e_ (red bar). ***B***, Ensemble average traces and mean values of 1 AP-stimulated exocytosis reported by vMAT2-pHluorin; **p < 0.01; ns, not significant by paired one-way ANOVA, Tukey’s *post hoc* test. ***C***, Schematic of the Ca^2+^ influx protocol with Fluo-5F (yellow bar) followed by washout and 1 AP stimulation with 4 mM [Ca^2+^]_e_ (gray bar), 0.7 mM isoflurane in 4 mM [Ca^2+^]_e_ (red bar) or 2 mM [Ca^2+^]_e_ (black bar). ***D***, Fluorescence microscopy image of VAMP-mCherry transfected neuron from which Ca^2+^ signals were recorded. Scale bar, 5 μm. ***E***, Fluorescence traces from individual boutons of the neuron in panel ***D*** with and without isoflurane in 4 mM [Ca^2+^]_e_. Mean responses are shown in bold. ***F***, Ensemble average traces. ***G***, Mean values of 1 AP-stimulated Ca^2+^ influx reported by Fluo-5F in 4 mM [Ca^2+^]_e_, isoflurane in 4 mM [Ca^2+^]_e_, 2 mM [Ca^2+^]_e_, or isoflurane in 2 mM [Ca^2+^]_e_(right); ***p < 0.001; ns, not significant by paired one-way ANOVA with Tukey’s *post hoc* test.

Isoflurane inhibits SV exocytosis in glutamatergic and GABAergic hippocampal neurons by reducing Ca^2+^ influx without affecting Ca^2+^ sensitivity indicated by the relationship between intracellular Ca^2+^ concentration and SV exocytosis (Baumgart et al., 2015). We examined this in dopaminergic neurons by comparing the effects of reduced extracellular Ca^2+^ concentration ([Ca^2+^]_e_) on SV exocytosis and Ca^2+^ influx using single AP stimuli to determine Ca^2+^ sensitivity (Fig. 7*A*,*C*). Isoflurane inhibited single AP-evoked SV exocytosis by 34 ± 6% (0.89 ± 0.19% of TP for 4 mM [Ca^2+^]_e_ control vs 0.58 ± 0.12% of TP with isoflurane; *p* = 0.005)^m^. This reduction in exocytosis was mimicked by reducing [Ca^2+^]_e_ from 4 mM to 2 mM in the absence of isoflurane. Exocytosis in 2 mM [Ca^2+^]_e_ was 0.51 ± 0.13% of TP (44 ± 5% reduction vs 4 mM [Ca^2+^]_e_), which was not significantly different from the inhibition by isoflurane in 4 mM [Ca^2+^]_e_ (*p* = 0.63; *n* = 6; Fig. 7*B*)^n^. A comparable effect was observed using the Ca^2+^ indicator Fluo-5F to measure changes in intracellular [Ca^2+^] (Fig. 7*D–G*), which is proportional to presynaptic Ca^2+^ influx (Hoppa et al., 2012). Isoflurane inhibited Ca^2+^ influx by 41 ± 3% in 4 mM Ca^2+^ (0.25 ± 0.02 ΔF/F_o_ for 4 mM [Ca^2+^]_e_ control vs 0.15 ± 0.02 ΔF/F_o_ with isoflurane; *p* = 0.0003)^o^. Presynaptic Ca^2+^ influx was inhibited to the same degree by reducing [Ca^2+^]_e_ from 4 mM to 2 mM: Ca^2+^ influx in 2 mM [Ca^2+^]_e_ was 0.15 ± 0.01 ΔF/F_o_ (36 ± 8% reduction vs 4 mM [Ca^2+^]_e_). Additionally, isoflurane inhibited Ca^2+^ influx to a similar degree in 2 mM Ca^2+^ (42 ± 3%; *p* = 0.0004)^p^, indicating the noncompetitive nature of isoflurane with respect to the ability of Ca^2+^ ions to flow through Ca^2+^ channels. There was no significant difference between Ca^2+^ influx with 2 mM [Ca^2+^]_e_ compared with 4 mM [Ca^2+^]_e_ plus isoflurane (*p* = 0.99; *n* = 5; Fig. 7*G*)^q^.

## Discussion

**Figure 8.**
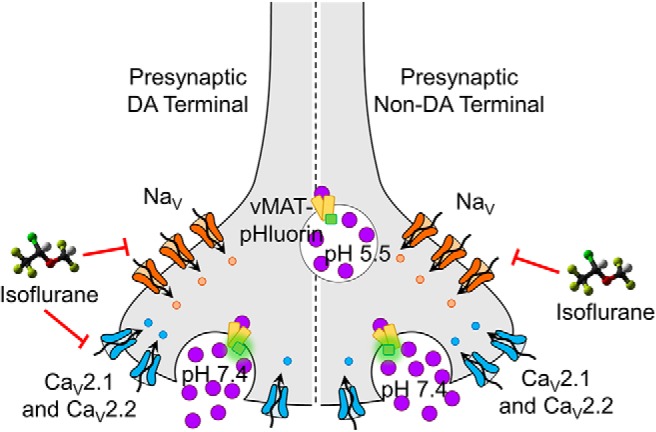
Presynaptic mechanisms of isoflurane. In non-dopaminergic (non-DA) neurons, isoflurane inhibits SV exocytosis by inhibiting Na^+^ channels to reduce nerve terminal excitability. In dopaminergic (DA) neurons, isoflurane can also reduce SV exocytosis by a Na^+^ channel-independent mechanism mediated by inhibition of Ca_V_2.1 and Ca_V_2.2. The fluorescence of vMAT2-pHluorin is quenched by the acidic (∼pH 5.5) SV interior. On exocytosis and fusion with the plasma membrane, vMAT2-pHluorin fluoresces on exposure to the pH neutral (∼pH 7.4) synaptic cleft.

Isoflurane inhibited SV exocytosis from cultured dopaminergic neurons by reducing Ca^2+^ entry though both Ca_V_2.1 and Ca_V_2.2 by a mechanism that is independent of Na^+^ channel activation (Fig. 8). This is in contrast to the predominant Na^+^ channel-dependent mechanism observed for release of glutamate or GABA in non-dopaminergic cortical and hippocampal neurons (Westphalen et al., 2010; Baumgart et al., 2015). These findings reveal important neurotransmitter-selective differences in the presynaptic mechanisms of isoflurane, a clinically essential volatile anesthetic. These differences provide a pharmacological rationale for developing novel anesthetics targeting specific anesthetic endpoints mediated by a single neurotransmitter system, for example dopaminergic control of emergence from unconsciousness.

### Relationship between Ca^2+^ channel subtypes and exocytosis

The Ca^2+^ channel subtypes present in dopaminergic neuron somata identified using whole-cell voltage-clamp recordings do not necessarily reflect the presynaptic Ca^2+^ channels involved in SV exocytosis. In rat dopaminergic midbrain neurons, somatic Ca^2+^ currents are inhibited by the L-type channel blocker nimodipine (by 28%), the Ca_V_2.2 blocker conotoxin (by 22%), and the Ca_V_2.1 blocker agatoxin (by 37%; Cardozo and Bean, 1995). However, synaptic boutons in rat dopaminergic neurons are too small for such direct electrophysiological recording of presynaptic Ca^2+^ currents. We used high-resolution live-cell imaging to measure SV exocytosis and Ca^2+^ influx, employing specific Ca^2+^ channel toxins to determine contributions of the major presynaptic Ca^2+^ channel subtypes. SV exocytosis in rat dopaminergic VTA neurons was mediated exclusively by Ca_V_2.1 and Ca_V_2.2, with Ca_V_2.1 predominating. Using KCl-induced depolarization to evoke SV exocytosis independent of Na^+^ channel involvement, isoflurane inhibited exocytosis mediated by either Ca_V_2.1 or Ca_V_2.2 to a similar degree, suggesting a lack of subtype selectivity. Alternatively, the effects of isoflurane are mediated via an unknown mechanism distinct to dopaminergic VTA neurons. One possibility is that K^+^ channel activity alters the resting membrane potential and therefore modifies open probability of Ca_V_2.1 and Ca_V_2.2.

Isoflurane inhibited SV exocytosis in dopaminergic neurons evoked by electrical stimulation of APs or elevated KCl depolarization, in contrast to non-dopaminergic neurons in which isoflurane inhibited AP-evoked but not KCl-evoked exocytosis. AP-evoked exocytosis requires activation of Na^+^ channels to sufficiently depolarize boutons and activate the presynaptic Ca^2+^ channels linked to exocytosis. Clamped depolarization by elevated KCl is less physiologic than repetitive electrical stimulation by causing sustained depolarization (Tibbs et al., 1989), which could alter Ca^2+^ channel relationship to exocytosis. This is suggested by the finding that with electrical stimulation conotoxin inhibited exocytosis in dopaminergic neurons by ∼40% and agatoxin inhibited by ∼80%, while with KCl-evoked depolarization both conotoxin and agatoxin inhibited exocytosis by 20–30%. The linkage between SV exocytosis to critical presynaptic Ca^2+^ channels in dopaminergic neurons is selectively sensitive to isoflurane, since isoflurane inhibition of KCl-evoked exocytosis was observed in dopaminergic, but not in non-dopaminergic, neurons. This fundamental neurotransmitter-specific difference in the relationship between Ca^2+^ channels and exocytosis results in profound neurotransmitter-specific differences in anesthetic sensitivity with potential neuropharmacological implications. This neurochemical difference is preserved in nigrostriatal dopaminergic nerve terminals prepared from rat striatum in which isoflurane also inhibits dopamine release via a Na^+^ channel-independent mechanism, an action that might contribute to the motor effects of anesthetics (Westphalen et al., 2013). The cellular and molecular attributes underlying this selective anesthetic pharmacology in dopaminergic neurons are unknown and await further characterization of the neurobiology of dopaminergic compared to non-dopaminergic neurons.

### Mechanisms of dopamine SV exocytosis

Monoamine neurotransmitters such as dopamine are packaged into both small SVs and large dense core vesicles (LDCVs) for release. The biosensor vMAT2-pHluorin labels both small SVs and LDCVs (Fei et al., 2008); however, only small SVs localize to presynaptic active zones of synaptic boutons for exocytosis, while LDCVs engage primarily in extrasynaptic exocytosis (Thureson-Klein, 1983; Südhof and Rizo, 2012). Moreover, the kinetics of SV exocytosis from small SVs and LDCVs are distinct: small SVs fuse within 1 ms of Ca^2+^ channel opening (Cohen et al., 1991), while LDCV fusion is 100-fold slower and therefore less tightly regulated to AP stimulation (Almers, 1990; Martin, 1994). Based on these characteristics, the SV exocytosis measured by the vMAT2-pHluorin method is primarily from small SVs (Pothos et al., 2000; Leenders et al., 2002). Anesthetic effects on asynchronous neuronal LDCV exocytosis might involve distinct mechanisms.

Neuroendocrine cells such as adrenal chromaffin cells or PC12 cells are frequently used to study catecholaminergic SV exocytosis but exhibit release mostly from LDCVs (Voets et al., 2001). This is an important distinction as the subcellular and molecular organization of the secretory machinery differ between dopaminergic neurons and neuroendocrine cells, which makes the latter poor models for midbrain neurons. For example, neuroendocrine cells do not have active release zones co-localized with Ca^2+^ microdomains, and the functional linkage of Ca^2+^ channels to release sites is not as tight as in neurons (Wu et al., 2009). Moreover, the Ca^2+^ channel subtypes linked to SV exocytosis differ between small SVs and LDCVs, with L-type channels closely linked to LDCV exocytosis (Park and Kim, 2009). In contrast, we found dopaminergic SV exocytosis to be independent of L-type Ca^2+^ channels.

### Differences between dopaminergic and non-dopaminergic neurons

When comparing the effects of isoflurane on dopaminergic and non-dopaminergic neurons it is important to consider that vMAT2 is not endogenously expressed in non-dopaminergic neurons (Yoo et al., 2016). However, vMAT2-pHluorin is still effective in measuring SV exocytosis in non-dopaminergic neurons due to ectopic expression. Transfection of vMAT2-pHluorin involves overexpression of vMAT2, which can increase quantal size, but this does not interfere with its use as an indicator of SV fusion (Pothos et al., 2000; Erickson et al., 2006).

In contrast to dopaminergic neurons, elevated KCl-evoked SV exocytosis from non-dopaminergic neurons was insensitive to isoflurane. This is consistent with previous observations of a Na_V_-dependent/Ca_v_-independent mechanism for rat glutamatergic and GABAergic hippocampal neurons (Hemmings et al., 2005a; Westphalen and Hemmings, 2006), despite their expression of both Ca_V_2.1 and Ca_V_2.2 (Qian and Noebels, 2001; Kamp et al., 2012). There are other important differences between dopaminergic and non-dopaminergic neurons that could explain their differential sensitivities to anesthetics. Non-dopaminergic neurons from the VTA are primarily GABAergic, some of which are capable of co-releasing glutamate (Carr and Sesack, 2000; Creed et al., 2014; Barker et al., 2016). In cultured rat hippocampal neurons, isoflurane inhibits SV exocytosis from glutamatergic boutons more potently than from GABAergic boutons due to a greater reduction in presynaptic Ca^2+^ influx (Baumgart et al., 2015), indicating that GABAergic neurons are less sensitive to isoflurane. This neuronal phenotypic difference in anesthetic sensitivity is consistent with the data presented here, as isoflurane more potently inhibited electrically-evoked SV exocytosis in dopaminergic neurons than in non-dopaminergic VTA neurons. It is likely that the non-dopaminergic VTA neurons were primarily GABAergic given their abundance in this nucleus. These neurotransmitter-specific differences in presynaptic sensitivity to isoflurane are likely due to differential presynaptic expression of specific ion channel subtypes (Johnson et al., 2017) with different anesthetic sensitivities that contribute to SV exocytosis.

Considering their numerous possible subunit compositions and splice variants, different ion channel subtypes and variants could contribute to presynaptic Ca^2+^ entry and SV release in different boutons (Meir et al., 1999). Differences in ion channel expression and degree of functional linkage (tight or loose) between Ca^2+^ entry and SV exocytosis also exist between various neuronal phenotypes (Eggermann et al., 2011). Differences in expression of various presynaptic Ca^2+^ binding proteins (e.g., calmodulin, present in all neurons, or calbindin, selectively expressed in some dopaminergic neurons) might also determine observed nerve terminal-specific differences in anesthetic sensitivity (Pan and Ryan, 2012). Further studies are necessary to determine the molecular specializations that underlie these presynaptic differences in anesthetic sensitivity.

### Role of dopaminergic neurons in general anesthesia

Mammalian dopaminergic neurons are located primarily in the substantia nigra pars compacta and the VTA. They project widely to forebrain regions including the dorsal striatum and nucleus accumbens, where dopamine release is essential to motor function and motivated behaviors, respectively (Wise, 2004). Electrical stimulation of the VTA, but not of the substantia nigra, facilitates emergence from isoflurane anesthesia in adult rats (Solt et al., 2014; Taylor et al., 2016). This effect is mediated by D1 dopamine receptors since selective pharmacological activation of D1, but not D2, receptors induces emergence from isoflurane anesthesia (Taylor et al., 2013). Moreover, the dopamine transporter (DAT) inhibitors methylphenidate and dextro-amphetamine restore conscious behaviors in rats anesthetized with isoflurane, propofol, or sevoflurane (Solt et al., 2011; Chemali et al., 2012; Kenny et al., 2015). Our findings that isoflurane inhibits SV exocytosis from midbrain dopamine neurons provides mechanistic support for the hypothesis that reduced dopamine signaling is associated with isoflurane-induced unconsciousness through reduced D1 receptor activation due to reduced dopamine exocytosis.

## Conclusions

Isoflurane inhibits SV exocytosis from rat dopaminergic neurons through direct inhibition of Ca^2+^ influx mediated by Ca_V_2.1 and/or Ca_V_2.2 independent of Na^+^ channel involvement. This neurotransmitter-selective presynaptic mechanism provides a molecular substrate for the role for dopaminergic VTA neurons in modulating isoflurane-induced unconsciousness. Improved understanding of these mechanisms is critical to elucidating how general anesthetics work to optimize their safe use and develop more specific drugs with fewer adverse effects.

**Table 1:** Statistical Data

	Data structure	Type of test	Confidence intervals
a	Normally distributed	Two-tailed paired *t* test	1.91 to 3.53
b	Normally distributed	Two-tailed paired *t* test	0.456 to 2.99
c	Normally distributed	One-tailed *t* test	–27.55 to –1.25
d	Normally distributed	One-way ANOVA Tukey’s *post hoc*	–3.968 to 3.116
e	Normally distributed	Two-tailed paired *t* test	2.13 to 5.11
f	Normally distributed	Two-tailed paired *t* test	–0.91 to 1.22
g	Normally distributed	One-way ANOVA Tukey’s *post hoc*	3.47 to 47.34
h	Normally distributed	One-way ANOVA Tukey’s *post hoc*	20.3 to 59.9
i	Normally distributed	One-way ANOVA Tukey’s *post hoc*	–28.97 to 18.66
j	Normally distributed	Two-tailed paired *t* test	17.26 to 29.82
k	Normally distributed	Two-tailed paired *t* test	6.35 to 37.58
l	Normally distributed	One-tailed *t* test	–23.96 to 32.65
m	Normally distributed	One-way ANOVA Tukey’s *post hoc*	0.115 to 0.515
n	Normally distributed	One-way ANOVA Tukey’s *post hoc*	–0.134 to 0.265
o	Normally distributed	Paired one-way ANOVA Tukey’s *post hoc*	0.071 to 0.135
p	Normally distributed	Paired one-way ANOVA Tukey’s *post hoc*	0.043 to 0.086
q	Normally distributed	Paired one-way ANOVA Tukey’s *post hoc*	–0.072 to 0.064

## Synthesis

Reviewing Editor: Katalin Toth, Universite Laval Decisions are customarily a result of the Reviewing Editor and the peer reviewers coming together and discussing their recommendations until a consensus is reached. When revisions are invited, a fact-based synthesis statement explaining their decision and outlining what is needed to prepare a revision will be listed below. The following reviewer(s) agreed to reveal their identity: NONE.

The current manuscript addresses how the volatile anesthetic isoflurane affects synaptic transmission in rat VTA dopaminergic neurons. The authors note significant differences between dopaminergic and non-dopaminergic neurons. The study is technically solid and the results support the authors' conclusions. The potential impact of the manuscripts should be increased by providing information on the following 3 main questions:

1. The authors should carry out a dose response curve to measure the calcium sensitivity of release and determine how the isoflurane impacts the Ca2+ sensitivity of release in dopaminergic neurons.

2. Anesthetic concentration: A dose-response relationship would be useful to determine the magnitude of the effect at lower doses of isoflurane that are sufficient to produce unconsciousness.

3. Please, elaborate in the text about the potential cell-types of the non-dopaminergic neurons.

Reviewers also identified few additional points that should be addressed by the authors:

Rev.#1

In this study the authors found that isoflurane significantly reduces presynaptic dopamine release from cultured rat VTA neurons, through direct inhibition of calcium influx mediated by P/Q-type or N-type calcium channels. Dopamine release from the VTA was previously shown to reverse the state of general anesthesia in rats and mice, and this study suggests that inhibition of dopamine release by VTA neurons may be one mechanism by which isoflurane produces unconsciousness. The manuscript is well written and easy to follow. Volatile anesthetics can be difficult to work with, but the authors took the appropriate steps to ensure that evaporative loss of isoflurane was minimized, and made the necessary calculations to account for the difference between experimental and physiological temperature. My main concern is that 2 MAC was used as the anesthetic concentration. This is higher than the dose required for unconsciousness, which is less than 1 MAC. Considering that the dose of anesthetic was quite high, the observed reduction in dopamine release was rather modest. A dose-response relationship would be useful to determine the magnitude of the effect at lower doses of isoflurane that are sufficient to produce unconsciousness.

Rev. #2.

1) The authors should provide images of the calcium imaging experiments and demonstrate that the Ca2+ signals were not contaminated by dendritic Ca2+ signals.

2) The results are based on averaged values and do not contain any single bouton analysis. It would be helpful to see some single bouton analysis for Figure 5 and Figure 6.

3) it needs to be specified how many different coverslips were used for each experiment. It is well known that primary cultured neurons can have artifacts due to culturing conditions. Therefore, it is important for the reader to know how many different coverslips and if they were from different batches of primary cultured neurons.

4) Data listed as data not shown in the manuscript. Should be shown.

5) N Values are not reported for Figure 5.

6) Values in all graphs should be shown as a bar graph with scatter plot, this will give a better approximation of the data. In addition, normalization graphs of Aga and Conotoxin sensitivity of the individual neurons in Figure 5 and 6 should be plotted.

7) The term coupling should be avoided in the manuscript. Coupling implies physical distance between channels and vesicles. This was not measured in this manuscript. It is better to say exocytosis being P/Q or N type dependent.

8) Please replace P/Q and N type labeling with current voltage gated calcium channel nomenclature Cav2.1 and Cav2.1 etc etc....

## References

[B1] Almers W (1990) Exocytosis. Annu Rev Physiol 52:607–624. 10.1146/annurev.ph.52.030190.003135 2184769

[B2] Anantharam A, Onoa B, Edwards RH, Holz RW, Axelrod D (2010) Localized topological changes of the plasma membrane upon exocytosis visualized by polarized TIRFM. J Cell Biol 188:415–428. 10.1083/jcb.200908010 20142424 PMC2819686

[B3] Ariel P, Hoppa MB, Ryan TA (2013) Intrinsic variability in Pv, RRP size, Ca(2+) channel repertoire, and presynaptic potentiation in individual synaptic boutons. Front Synaptic Neurosci 4:9. 10.3389/fnsyn.2012.00009 23335896 PMC3542534

[B4] Atluri PP, Ryan TA (2006) The kinetics of synaptic vesicle reacidification at hippocampal nerve terminals. J Neurosci 26:2313–2320. 10.1523/JNEUROSCI.4425-05.2006 16495458 PMC6674811

[B5] Barker DJ, Root DH, Zhang S, Morales M (2016) Multiplexed neurochemical signaling by neurons of the ventral tegmental area. J Chem Neuroanat 73:33–42. 10.1016/j.jchemneu.2015.12.016 26763116 PMC4818729

[B6] Barrot M (2014) The ventral tegmentum and dopamine: a new wave of diversity. Neuroscience 282:243–247. 10.1016/j.neuroscience.2014.10.017 25453764

[B7] Baumgart JP, Zhou ZY, Hara M, Cook DC, Hoppa MB, Ryan TA, Hemmings HC Jr (2015) Isoflurane inhibits synaptic vesicle exocytosis through reduced Ca2+ influx, not Ca2+-exocytosis coupling. Proc Natl Acad Sci USA 112:11959–11964. 10.1073/pnas.1500525112 26351670 PMC4586856

[B8] Bosnjak ZJ, Supan FD, Rusch NJ (1991) The effects of halothane, enflurane, and isoflurane on calcium current in isolated canine ventricular cells. Anesthesiology 74:340–345. 10.1097/00000542-199102000-00022 1846726

[B9] Brown EN, Purdon PL, Van Dort CJ (2011) General anesthesia and altered states of arousal: a systems neuroscience analysis. Annu Rev Neurosci 34:601–628. 10.1146/annurev-neuro-060909-153200 21513454 PMC3390788

[B10] Cardozo DL, Bean BP (1995) Voltage-dependent calcium channels in rat midbrain dopamine neurons: modulation by dopamine and GABAB receptors. J Neurophysiol 74:1137–1148. 10.1152/jn.1995.74.3.1137 7500139

[B11] Carr DB, Sesack SR (2000) GABA-containing neurons in the rat ventral tegmental area project to the prefrontal cortex. Synapse 38:114–123. 10.1002/1098-2396(200011)38:2<114::aid-syn2>3.0.co;2-r 11018785

[B12] Chemali JJ, Van Dort CJ, Brown EN, Solt K (2012) Active emergence from propofol general anesthesia is induced by methylphenidate. Anesthesiology 116:998–1005. 10.1097/ALN.0b013e3182518bfc 22446983 PMC3339625

[B13] Creed MC, Ntamati NR, Tan KR (2014) VTA GABA neurons modulate specific learning behaviors through the control of dopamine and cholinergic systems. Front Behav Neurosci 8:8. 10.3389/fnbeh.2014.00008 24478655 PMC3897868

[B14] Cohen MW, Jones OT, Angelides KJ (1991) Distribution of Ca2+ channels on frog motor nerve terminals revealed by fluorescent omega-conotoxin. J Neurosci 11:1032–1039. 10.1523/jneurosci.11-04-01032.1991 1707093 PMC6575372

[B15] Eggermann E, Bucurenciu I, Goswami SP, Jonas P (2011) Nanodomain coupling between Ca2+ channels and Ca2+ sensors of exocytosis at fast mammalian synapses. Nat Rev Neurosci 13:7–21. 10.1038/nrn3125 22183436 PMC3617475

[B16] Erickson JD, De Gois S, Varoqui H, Schafer MK, Weihe E (2006) Activity-dependent regulation of vesicular glutamate and GABA transporters: a means to scale quantal size. Neurochem Int 48:643–649. 10.1016/j.neuint.2005.12.029 16546297

[B17] Evans RM, Zamponi GW (2006) Presynaptic Ca2+ channels–integration centers for neuronal signaling pathways. Trends Neurosci 29:617–624. 10.1016/j.tins.2006.08.006 16942804

[B18] Fei H, Grygoruk A, Brooks ES, Chen A, Krantz DE (2008) Trafficking of vesicular neurotransmitter transporters. Traffic 9:1425–1436. 10.1111/j.1600-0854.2008.00771.x 18507811 PMC2897747

[B19] Franks NP, Lieb WR (1993) Selective actions of volatile general anaesthetics at molecular and cellular levels. Br J Anaesth 71:65–76. 10.1093/bja/71.1.65 7688242

[B20] Goetze B, Grunewald B, Baldassa S, Kiebler M (2004) Chemically controlled formation of a DNA/calcium phosphate coprecipitate: application for transfection of mature hippocampal neurons. J Neurobiol 60:517–525. 10.1002/neu.20073 15307155

[B21] Hall AC, Lieb WR, Franks NP (1994) Insensitivity of P-type calcium channels to inhalational and intravenous general anesthetics. Anesthesiology 81:117–123. 10.1097/00000542-199407000-00017 8042779

[B23] Hemmings HC Jr, Yan W, Westphalen RI, Ryan TA (2005a) The general anesthetic isoflurane depresses synaptic vesicle exocytosis. Mol Pharmacol 67:1591–1599. 10.1124/mol.104.003210 15728262

[B24] Hemmings HC, Akabas MH, Goldstein PA, Trudell JR, Orser BA, Harrison NL (2005b) Emerging molecular mechanisms of general anesthetic action. Trends Pharmacol Sci 26:503–510. 10.1016/j.tips.2005.08.006 16126282

[B25] Hoppa MB, Lana B, Margas W, Dolphin AC, Ryan TA (2012) α2δ expression sets presynaptic calcium channel abundance and release probability. Nature 486:122–125. 10.1038/nature11033 22678293 PMC3376018

[B26] Jiang M, Chen G (2006) High Ca2+-phosphate transfection efficiency in low-density neuronal cultures. Nat Protoc 1:695–700. 10.1038/nprot.2006.86 17406298

[B27] Johnson KW, Herold KF, Milner TA, Hemmings HC Jr, Platholi J (2017) Sodium channel subtypes are differentially localized to pre- and post-synaptic sites in rat hippocampus. J Comp Neurol 525:3563–3578. 10.1002/cne.24291 28758202 PMC5927368

[B28] Joksovic PM, Weiergräber M, Lee W, Struck H, Schneider T, Todorovic SM (2009) Isoflurane-sensitive presynaptic R-type calcium channels contribute to inhibitory synaptic transmission in the rat thalamus. J Neurosci 29:1434–1445. 10.1523/JNEUROSCI.5574-08.2009 19193890 PMC2659547

[B29] Kamp MA, Hänggi D, Steiger HJ, Schneider T (2012) Diversity of presynaptic calcium channels displaying different synaptic properties. Rev Neurosci 23:179–190. 10.1515/revneuro-2011-0070 22499676

[B30] Kenny JD, Taylor NE, Brown EN, Solt K (2015) Dextroamphetamine (but not atomoxetine) induces reanimation from general anesthesia: implications for the roles of dopamine and norepinephrine in active emergence. PLoS One 10:e0131914. 10.1371/journal.pone.0131914 26148114 PMC4492624

[B31] Leenders AG, Hengst P, Lopes da Silva FH, Ghijsen WE (2002) A biochemical approach to study sub-second endogenous release of diverse neurotransmitters from central nerve terminals. J Neurosci Methods 113:27–36. 10.1016/s0165-0270(01)00472-1 11741718

[B32] Liu C, Kershberg L, Wang J, Schneeberger S, Kaeser PS (2018) Dopamine secretion is mediated by sparse active zone-like release sites. Cell 172:706–718. 10.1016/j.cell.2018.01.008 29398114 PMC5807134

[B33] Lynch C 3rd, Vogel S, Sperelakis N (1981) Halothane depression of myocardial slow action potentials. Anesthesiology 55:360–368. 10.1097/00000542-198110000-00005 7294370

[B34] Martin TF (1994) The molecular machinery for fast and slow neurosecretion. Curr Opin Neurobiol 4:626–632. 10.1016/0959-4388(94)90002-7 7849517

[B35] Meir A, Ginsburg S, Butkevich A, Kachalsky SG, Kaiserman I, Ahdut R, Demirgoren S, Rahamimoff R (1999) Ion channels in presynaptic nerve terminals and control of transmitter release. Physiol Rev 79:1019–1088. 10.1152/physrev.1999.79.3.1019 10390521

[B36] Mena MA, Khan U, Togasaki DM, Sulzer D, Epstein CJ, Przedborski S (1997) Effects of wild-type and mutated copper/zinc superoxide dismutase on neuronal survival and L-DOPA-induced toxicity in postnatal midbrain culture. J Neurochem 69:21–33. 10.1046/j.1471-4159.1997.69010021.x 9202290

[B37] Monti JM, Monti D (2007) The involvement of dopamine in the modulation of sleep and waking. Sleep Med Rev 11:113–133. 10.1016/j.smrv.2006.08.003 17275369

[B38] Murakami N, Ishibashi H, Katsurabayashi S, Akaike N (2002) Calcium channel subtypes on single GABAergic presynaptic terminal projecting to rat hippocampal neurons. Brain Res 951:121–129. 10.1016/s0006-8993(02)03148-7 12231465

[B39] Ouyang W, Hemmings HC Jr (2005) Depression by isoflurane of the action potential and underlying voltage-gated ion currents in isolated rat neurohypophysial nerve terminals. J Pharmacol Exp Ther 312:801–808. 10.1124/jpet.104.074609 15375177

[B40] Pan PY, Ryan TA (2012) Calbindin controls release probability in ventral tegmental area dopamine neurons. Nat Neurosci 15:813–815. 10.1038/nn.3099 22544312 PMC3703651

[B41] Park Y, Kim KT (2009) Dominant role of lipid rafts L-type calcium channel in activity-dependent potentiation of large dense-core vesicle exocytosis. J Neurochem 110:520–529. 10.1111/j.1471-4159.2009.06148.x 19457106

[B42] Pothos EN, Larsen KE, Krantz DE, Liu Y, Haycock JW, Setlik W, Gershon MD, Edwards RH, Sulzer D (2000) Synaptic vesicle transporter expression regulates vesicle phenotype and quantal size. J Neurosci 20:7297–7306. 10.1523/JNEUROSCI.20-19-07297.2000 11007887 PMC6772799

[B43] Qian J, Noebels JL (2001) Presynaptic Ca2+ channels and neurotransmitter release at the terminal of a mouse cortical neuron. J Neurosci 21:3721–3728. 10.1523/jneurosci.21-11-03721.2001 11356859 PMC6762720

[B44] Ratnakumari L, Hemmings HC Jr (1998) Inhibition of presynaptic sodium channels by halothane. Anesthesiology 88:1043–1054. 10.1097/00000542-199804000-00025 9579514

[B45] Reid CA, Clements JD, Bekkers JM (1997) Nonuniform distribution of Ca^2+^ channel subtypes on presynaptic terminals of excitatory synapses in hippocampal cultures. J Neurosci 17:2738–2745. 10.1523/jneurosci.17-08-02738.1997 9092595 PMC6573101

[B46] Rudolph U, Antkowiak B (2004) Molecular and neuronal substrates for general anaesthetics. Nat Rev Neurosci 5:709–720. 10.1038/nrn1496 15322529

[B47] Sankaranarayanan S, De Angelis D, Rothman JE, Ryan TA (2000) The use of pHluorins for optical measurements of presynaptic activity. Biophys J 79:2199–2208. 10.1016/S0006-3495(00)76468-X 11023924 PMC1301110

[B48] Solt K, Cotten JF, Cimenser A, Wong KF, Chemali JJ, Brown EN (2011) Methylphenidate actively induces emergence from general anesthesia. Anesthesiology 115:791–803. 10.1097/ALN.0b013e31822e92e5 21934407 PMC3178041

[B49] Solt K, Van Dort CJ, Chemali JJ, Taylor NE, Kenny JD, Brown EN (2014) Electrical stimulation of the ventral tegmental area induces reanimation from general anesthesia. Anesthesiology 121:311–319. 10.1097/ALN.0000000000000117 24398816 PMC4112744

[B50] Study RE (1994) Isoflurane inhibits multiple voltage-gated calcium currents in hippocampal pyramidal neurons. Anesthesiology 81:104–116. 10.1097/00000542-199407000-00016 8042778

[B51] Südhof TC, Rizo J (2012) Synaptic vesicle exocytosis. Cold Spring Harb Perspect Biol 3:a005637. 10.1101/cshperspect.a005637 22026965 PMC3225952

[B52] Taylor NE, Chemali JJ, Brown EN, Solt K (2013) Activation of D1 dopamine receptors induces emergence from isoflurane general anesthesia. Anesthesiology 118:30–39. 10.1097/ALN.0b013e318278c896 23221866 PMC3527840

[B53] Taylor NE, Van Dort CJ, Kenny JD, Pei J, Guidera JA, Vlasov KY, Lee JT, Boyden ES, Brown EN, Solt K (2016) Optogenetic activation of dopamine neurons in the ventral tegmental area induces reanimation from general anesthesia. Proc Natl Acad Sci USA 113:12826–12831. 10.1073/pnas.1614340113 27791160 PMC5111696

[B54] Thureson-Klein A (1983) Exocytosis from large and small dense cored vesicles in noradrenergic nerve terminals. Neuroscience 10:245–259. 10.1016/0306-4522(83)90132-x 6633860

[B55] Tibbs GR, Barrie AP, Van Mieghem FJ, McMahon HT, Nicholls DG (1989) Repetitive action potentials in isolated nerve terminals in the presence of 4-aminopyridine: effects on cytosolic free Ca2+ and glutamate release. J Neurochem 53:1693–1699. 10.1111/j.1471-4159.1989.tb09232.x 2553862

[B56] Voets T, Moser T, Lund PE, Chow RH, Geppert M, Südhof TC, Neher E (2001) Intracellular calcium dependence of large dense-core vesicle exocytosis in the absence of synaptotagmin I. Proc Natl Acad Sci USA 98:11680–11685. 10.1073/pnas.201398798 11562488 PMC58789

[B57] Westphalen RI, Hemmings HC Jr (2003) Selective depression by general anesthetics of glutamate versus GABA release from isolated cortical nerve terminals. J Pharmacol Exp Ther 304:1188–1196. 10.1124/jpet.102.044685 12604696

[B58] Westphalen RI, Hemmings HC Jr (2006) Volatile anesthetic effects on glutamate versus GABA release from isolated rat cortical nerve terminals: 4-aminopyridine-evoked release. J Pharmacol Exp Ther 316:216–223. 10.1124/jpet.105.090662 16174800

[B59] Westphalen RI, Yu J, Krivitski M, Jih TY, Hemmings HC Jr (2010) Regional differences in nerve terminal Na+ channel subtype expression and Na+ channel-dependent glutamate and GABA release in rat CNS. J Neurochem 113:1611–1620. 10.1111/j.1471-4159.2010.06722.x 20374421 PMC2914626

[B60] Westphalen RI, Desai KM, Hemmings HC Jr (2013) Presynaptic inhibition of the release of multiple major central nervous system neurotransmitter types by the inhaled anaesthetic isoflurane. Br J Anaesth 110:592–599. 10.1093/bja/aes448 23213036 PMC3600942

[B61] Wheeler DB, Randall A, Tsien RW (1994) Roles of N-type and Q-type Ca2+ channels in supporting hippocampal synaptic transmission. Science 264:107–111. 10.1126/science.7832825 7832825

[B62] White IL, Franks NP, Dickinson R (2005) Effects of isoflurane and xenon on Ba^2+^-currents mediated by N-type calcium channels. Br J Anaesth 94:784–790. 10.1093/bja/aei126 15778267

[B63] Wise RA (2004) Dopamine, learning and motivation. Nat Rev Neurosci 5:483–494. 10.1038/nrn1406 15152198

[B64] Wu LG, Westenbroek RE, Borst JG, Catterall WA, Sakmann B (1999) Calcium channel types with distinct presynaptic localization couple differentially to transmitter release in single calyx-type synapses. J Neurosci 19:726–736. 10.1523/JNEUROSCI.19-02-00726.1999 9880593 PMC6782194

[B65] Wu MM, Llobet A, Lagnado L (2009) Loose coupling between calcium channels and sites of exocytosis in chromaffin cells. J Physiol 587:5377–5391. 10.1113/jphysiol.2009.176065 19752110 PMC2793871

[B66] Wu XS, Sun JY, Evers AS, Crowder M, Wu LG (2004) Isoflurane inhibits transmitter release and the presynaptic action potential. Anesthesiology 100:663–670. 10.1097/00000542-200403000-00029 15108983

[B67] Yoo JH, Zell V, Gutierrez-Reed N, Wu J, Ressler R, Shenasa MA, Johnson AB, Fife KH, Faget L, Hnasko TS (2016) Ventral tegmental area glutamate neurons co-release GABA and promote positive reinforcement. Nat Commun 7:13697. 10.1038/ncomms13697 27976722 PMC5171775

[B68] Zhang Y, Sonner JM, Eger EI 2nd, Stabernack CR, Laster MJ, Raines DE, Harris RA (2004) Gamma-aminobutyric acidA receptors do not mediate the immobility produced by isoflurane. Anesth Analg 99:85–90. 10.1213/01.ANE.0000118108.64886.42 15281509

